# Identifying predictive features of *Clostridium difficile* infection recurrence before, during, and after primary antibiotic treatment

**DOI:** 10.1186/s40168-017-0368-1

**Published:** 2017-11-13

**Authors:** Sepideh Pakpour, Amit Bhanvadia, Roger Zhu, Abhimanyu Amarnani, Sean M. Gibbons, Thomas Gurry, Eric J. Alm, Laura A. Martello

**Affiliations:** 1 Genome Sequencing and Analysis Program, Broad Institute, Cambridge, MA USA; 2 Department of Biological Engineering, Massachusetts Institute of Technology (MIT), Cambridge, MA USA; 3 MIT Center for Microbiome Informatics and Therapeutics, Cambridge, MA USA; 4 Division of Digestive Diseases, Lenox Hill Hospital/Northwell Health, New York, NY USA; 5 Surgery, NewYork-Presbyterian/Queens, Flushing, NY USA; 6 Medicine, SUNY Downstate Medical Center, Brooklyn, NY USA

## Abstract

**Background:**

Colonization by the pathogen *Clostridium difficile* often occurs in the background of a disrupted microbial community. Identifying specific organisms conferring resistance to invasion by *C. difficile* is desirable because diagnostic and therapeutic strategies based on the human microbiota have the potential to provide more precision to the management and treatment of *Clostridium difficile* infection (CDI) and its recurrence.

**Methods:**

We conducted a longitudinal study of adult patients diagnosed with their first CDI. We investigated the dynamics of the gut microbiota during antibiotic treatment, and we used microbial or demographic features at the time of diagnosis, or after treatment, to predict CDI recurrence. To check the validity of the predictions, a meta-analysis using a previously published dataset was performed.

**Results:**

We observed that patients’ microbiota “before” antibiotic treatment was predictive of disease relapse, but surprisingly, post-antibiotic microbial community is indistinguishable between patients that recur or not. At the individual OTU level, we identified *Veillonella dispar* as a candidate organism for preventing CDI recurrence; however, we did not detect a corresponding signal in the conducted meta-analysis.

**Conclusion:**

Although in our patient population, a candidate organism was identified for negatively predicting CDI recurrence, results suggest the need for larger cohort studies that include patients with diverse demographic characteristics to generalize species that robustly confer colonization resistance against *C. difficile* and accurately predict disease relapse.

**Electronic supplementary material:**

The online version of this article (10.1186/s40168-017-0368-1) contains supplementary material, which is available to authorized users.

## Background

*Clostridium difficile* infection (CDI) is an urgent public health priority worldwide [1–5], and despite progress in infection control and innovative options for treatment of CDI, until recently, there has been a steady and considerable elevation in its incidence, as well as its reported severity of illness [2, 6–9]. Known factors associated with CDI include hospitalization, advanced age, antibiotic prescription, and gastrointestinal surgery, in addition to those less agreed upon such as proton pump inhibitor therapy [6, 9–14]. Standard management of CDI involves the administration of antibiotic therapy, such as metronidazole and vancomycin [15], but 22.4 and 14.2% of patients have been observed to have no response to metronidazole and vancomycin, respectively [16]. Of the remaining patients with positive responses to antibiotic therapy, 30% have shown CDI relapses [7, 15, 17]. CDI relapses add a layer of complexity to CDI management, and currently available clinical models have limited power to predict the risk of recurrence, either before or after discontinuation of *C. difficile* treatment.

For patients with multiple failures of antibiotic treatment for recurrent infection, fecal microbiota transplantation (FMT) has become an effective treatment strategy (with 92% success rate [18]). FMT, which was performed as early as the fourth century [19], aims to restore normal microbiota [20], highlighting the crucially important role of the gut microbiome in providing *C. difficile* colonization resistance. Studies have shown that gut dysbiosis leads to reduced colonization resistance against *C. difficile* and ultimately increases susceptibility to CDI [21, 22]. More specifically, 16S rRNA gene sequence analyses have demonstrated that the bacterial diversity of patients with initial and recurrent CDI is noticeably lower than that of healthy subjects [23, 24]. Furthermore, higher relative abundances of Proteobacteria and Firmicutes phyla, along with a lower relative abundance of Bacteroidetes, have been reported in CDI patients [25]. Researchers have begun investigating which specific bacterial signatures may be associated with CDI recurrence after treatment. For example, studies have associated elevated abundance of Enterobacteriaceae with increased susceptibility to recurrence [25–27]. More recently, positive associations between *Veillonella*, *Streptococcus*, *Parabacteroides*, and Lachnospiraceae and CDI recurrence have been suggested [22, 28].

Although the role of the gut microbiome in CDI susceptibility has been well established, the particular species contributing to recurrence likelihood remain unclear. Also, despite significant progress in our understanding of CDI and its recurrence, most studies have focused on static (single time point) features of the microbiome. The dynamics of the gut microbiome *during* treatment, and the association of these dynamic features with clinical/demographic factors, CDI severity, and recurrence, have not yet been scrutinized. Here, to fulfill the above gaps, we conducted the first prospective study along with a meta-analysis to uncover microbial signatures to predict recurrent CDI. Specific questions included: (1) are there any associations between severity of CDI, microbial or demographic features, and CDI recurrence and (2) can we use microbial or demographic features at the time of diagnosis, or after treatment, to predict CDI recurrence? The meta-analysis was done between our dataset and a recently published dataset [22] with similar sample collection, DNA extraction, primer selection, and sequencing methods. By applying standardized bioinformatics and statistical methods to these two independent studies, we aim to identify consistent biological signatures of CDI recurrence. These signatures may be useful targets for clinical diagnostics, helping to direct more effective treatments (e.g., FMT) to patients with a higher risk for CDI recurrence.

## Methods

### Study design and sample collection

Eligible male and female patient participants were identified at the State University of New York Downstate Medical Center (University Hospital of Brooklyn) and Kings County Hospital Center, Brooklyn, New York. Criteria for participation included *Clostridium difficile* infection (CDI) with clinically significant diarrhea symptoms (change in bowel movement habits with three or more liquid or uniformed stools within 24 h) along with confirmation by a positive laboratory stool test via stool polymerase chain reaction (PCR) or toxin B assays, as well as willingness to participate and the ability to maintain close follow-ups. All subjects signed an informed consent form prior to enrollment. Exclusion criteria for participation included history of inflammatory bowel disease (Crohn’s disease or ulcerative colitis) and total or subtotal colectomy.

A total of 31 individuals experiencing their first episode of CDI (median age 64.0 years, interquartile range 60.0–73.0; 51.6% female) were enrolled between March 2014 and April 2015. Participants were followed at regular intervals beginning at the time of diagnosis before the administration of antibiotics treatment (pre-treatment, *n* = 31), 2 days after the start of antibiotics treatment (post-treatment, *n* = 31), 7 days after the start of antibiotics treatment or at the time of discharge (whichever was earlier (pre-discharge, *n* = 18)), followed by the fourth stool samples collected 2 weeks after the start of antibiotics (4 days after treatment completion, post-discharge, *n* = 9). Severity of CDIs were assessed early in the course of the disease to adapt medical management using the University of Pittsburgh Medical Center (UPMC) Index (version 2) [29, 30], where a UPMC score lower than 2 indicated moderate CDI, and a score equal or greater than 2 indicated severe CDI. Treatment regimens for each patient were based on the Infectious Diseases Society of America (IDSA) guidelines. Specifically, vancomycin was used for patients having UPMC score equal or greater than 2, demonstrating signs of systemic toxicity with or without profuse diarrhea, or warranting an ICU admission. The rest of patients were treated with metronidazole.

At each sampling point, stool specimens were collected in standard specimen containers, aliquoted with sterile technique into RNAlater, and were flash-frozen at − 80 °C. We also included five stool samples from healthy donors, in order to compare diversity and composition of healthy subjects with patient participants.

Patient characteristics and clinical data including age, sex, diet, weight, height, immunosuppressive therapy, hospitalization within 3 months prior to CDI diagnosis, antibiotic treatment within 3 month prior to CDI diagnosis, PPI therapy, and ICU stay prior to CDI diagnosis, in addition to detailed laboratory metadata at the initial encounter were extracted from medical records and patient interviews. Two to 4 weeks after discharge, follow-up data including CDI treatment regimen and its recurrence were obtained. This protocol was approved by the Institutional Review Board (IRB) at State University of New York Downstate Medical Center and the Massachusetts Institute of Technology.

### DNA extraction and sequencing protocols

Total genomic DNA was extracted from 500-mg stool samples using the PowerFecal DNA Isolation kit (Mo Bio Laboratories, Carlsbad, CA, USA), according to the manufacturer’s instructions with the following modifications to improve yields from difficult-to-lyse bacteria. An additional bead-beating step using Faster Prep FP120 (Thermo) at 6 m/s for 1 min was used instead of vortex agitation. Incubation with buffers C2 and C3 was increased to 10 min at 4 °C. Subsequently, quantity of extracted DNA samples were measured by a Qubit Fluorometer (Life Technologies, Carlsbad, CA, USA), and then, extracted DNA of samples were sent to MIT-BioMicroCenter for multiplexed amplicon library preparation, covering the 16S rRNA gene V4 region using a dual-index barcode protocol, followed by Illumina MiSeq 16S rRNA gene sequencing.

### 16S rRNA gene data analysis

Sequencing of the stool samples on Illumina MiSeq instrument generated 7,176,335 total raw sequencing reads. Raw reads were processed using the QIIME version 1.8.0 [31] and custom Python scripts. Forward and reverse Illumina reads were joined, quality trimmed to a minimum PHRED score of 25, and then truncated to a length of 250; the lengths determined based on the mode of the read length distribution. Singleton reads were removed from the dataset, and chimeras were eliminated using the UPARSE-OTU algorithm [32]. Closed reference OTU picking was employed by aligning unique reads to the GreenGenes OTU database, at 99% identity (May 2013 release) using the USEARCH algorithm [33, 34]. Representative sequences for each OTU were aligned using PyNast, with a minimum alignment overlap of 75 bp [35], and a phylogenetic tree was built using FastTree 2.0 [36]. Of the 89 collected stool samples, a total of 6,754,571 high-quality sequence reads were identified, representing 6160 OTUs for downstream analyses. Relative abundances of different bacterial genera were obtained by collapsing 16S rRNA gene OTU taxonomies to the genus level and summing OTUs within the same genus. Finally, abundances of different bacterial families were obtained by collapsing 16S rRNA gene OTU taxonomies to the family level and summing OTUs within the same family. At each level, taxa occurring in only one sample as well as low abundance taxa, accounting for less than 0.05% of the total community were removed. This step reduced the total number of statistical tests that were performed and thus reduced the burden of multiple hypothesis testing. After filtering, 195 OTUs, 51 genera, and 16 families remained for downstream analyses.

### Statistical analysis

Severity of CDIs were assessed early in the course of the disease to adapt medical management using the University of Pittsburgh Medical Center (UPMC) Index (version 2) [29], where a UPMC score equal or greater than 2 indicated severe CDI. Microbial relationships between disease severity index, infection recurrence, and other collected metadata were evaluated by Spearman correlation with a false discovery rate (FDR) correction.

Overall microbial community diversity (*α*-diversity) was measured using the Shannon entropy [18, 21, 22]. Significant difference in *α*-diversity between groups (patients with and without recurrence) was determined using the Mann-Whitney *U* test. Differences in community structure across samples (*β*-diversity) were calculated using the weighted UniFrac distance metric and visualized by Principal Coordinates Analysis (PCoA) plots using custom R scripts. Significant differences in *β*-diversity across patient groups were evaluated using Permutational Multivariate Analysis of Variance (PERMANOVA) with 10^4^ permutations. We also performed Kruskal-Wallis tests using R between features of groups with and without recurrence. All *p* values were then adjusted using the FDR correction.

To test whether microbial community composition can predict recurrence after full treatment, we trained a random forest model on pre-treatment samples, at OTU, genus, and family levels. We evaluated their performance using leave-one-out cross-validation and scored the predictive power in a receiver operating characteristic (ROC) analysis. The discriminatory power of OTUs, genera, and families were calculated as the area under the ROC curve (AUC). To assess the random forest model constructed, study groups were shuffled randomly and 100 random forest classifications were computed. The out-of-bag error estimate was compared to the un-shuffled dataset using a one-sample Wilcoxon signed-rank test to assess the performance of the classification model.

### Meta-analysis

To further check the validity of the prediction results, a meta-analysis was performed using recent data published by Khanna et al. [22], which also aimed to find microbial fingerprints predicting the risk of recurrence after successful treatment in patients with primary CDI (more information on both studies can be found in Table 1).

**Table 1:** Characteristics of the studies

Study	Target region	Sequence platform	DNA extraction protocol	Patients’ age	Patients’ sex (%)	BMI (kg/m^2^)
Khanna et al. [22]	V4	MiSeq Illumina	PowerFecal DNA Isolation kit (Mo Bio)	Median, 52.7 Lower percentile, 36.9 Upper percentile, 65.1	M, 39.8 F, 60.2	Median, 26.7 Lower percentile, 23.1 Upper percentile, 30.6
Pakpour et al. (current)	V4	MiSeq Illumina	PowerFecal DNA Isolation kit (Mo Bio)	Median 64.0 Lower percentile, 60.0 Upper percentile, 73.0	M, 48.4 F, 51.6	Median, 25.0 Lower percentile, 20.4 Upper percentile, 31.7

Sequence data and sample metadata, shared by the original authors, were downloaded from the NCBI Sequence Read Archive (SRA, accession number: SRP087648). For the Khanna et al. [22] dataset, only patients that responded to primary treatment (with and without recurrence) were kept for the meta-analysis. In our dataset, because individuals were sampled at multiple time intervals, only samples at the pre-treatment stage were included in order to make the two datasets comparable. Also, because only forward reads were used in the Khanna et al. study, we also included only forward reads from our study in the meta-analysis. For each dataset, sequence reads were demultiplexed, followed by quality filtering (PHRED score of 25) and removing any reads containing ambiguous bases. For both studies, read lengths were truncated to 200 bp. Subsequently, the quality-filtered reads were pooled, followed OTU calling against a reference set of OTUs assembled at 99% similarity from the Greengenes database (May 2013 release), as described above. Tables at OTU, genus, and family levels were constructed, and finally at each level, low abundant taxa (covering < 0.05% of total OTUs) were removed.

*β*-Diversity was calculated using the weighted UniFrac distance metric, and significant differences across patient groups were evaluated using PERMANOVA. For predictive models, we trained a random forest model on each individual dataset as well as the combined (meta) dataset. We also built the model by training on one dataset and using the other for cross-validation. The discriminatory power of OTUs, genera, and families were calculated as the area under the ROC curve (AUC) in each case.

## Results

**Fig. 1:**
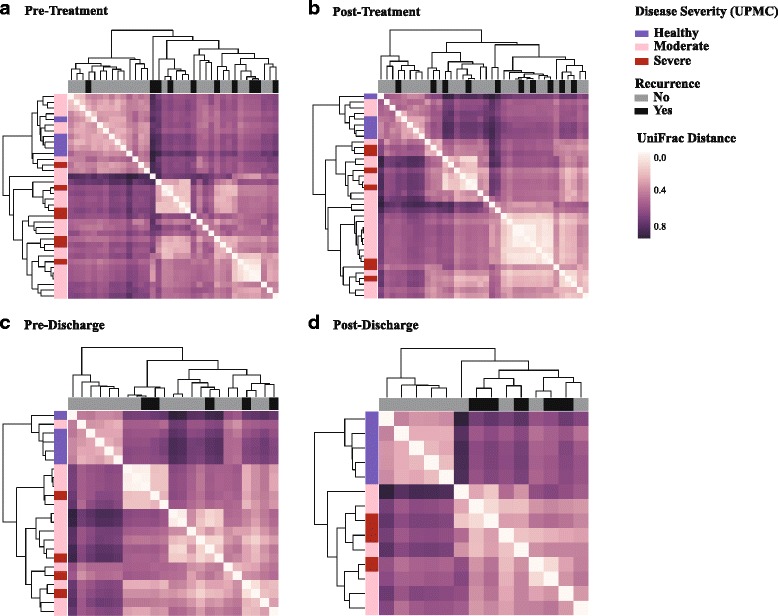
Hierarchically clustered heatmaps showing weighted UniFrac distances (*β*-diversity) between patient samples prior to antibiotic treatment (**a**), following antibiotic treatment (**b**), prior to discharge from hospital (**c**), and following discharge from hospital (**d**). Light purple indicates samples that are similar to one another, while dark purple shows highly dissimilar samples. The colored bars next to each row indicate disease severity (healthy, moderate CDI, and severe CDI). Colored bars above columns indicate CDI recurrence

This longitudinal study enrolled 31 patients experiencing their first episode of *Clostridium difficile* infection (CDI), seven of whom met the criteria of severe or complicated disease (UPMC score ≥ 2 [29]). The patient population was mostly of Afro-Caribbean descent, and 54.8% of them were taking proton pump inhibitors (Additional file 1). 16S rRNA gene analyses at the pre-treatment level revealed random clustering of moderately infected patients with both healthy individuals and those with severe disease (Fig. 1a). However, gut microbial communities in patients with severe infection were significantly dissimilar when compared to healthy individuals (PERMANOVA, *p* value = 0.004). Over the course of antibiotic treatment, gut microbial community structures in infected patients (moderate and severe) became gradually more similar to each other, with greater distance from controls (Fig. 1b, c); the most distinct clusters were observed at the post-discharge stage (Fig. 1d). Follow-up data confirmed infection recurrence in 32% of patients with no significant relationship with disease severity index or any other metadata variable (e.g., age, sex, BMI, pre-CDI antibiotic therapy, PPI) as determined by FDR-corrected Spearman correlations.

**Fig. 2:**
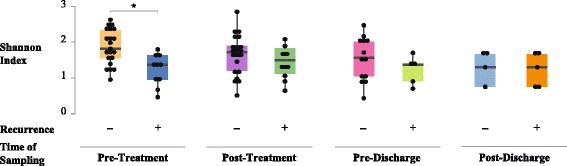
Boxplots show distributions of Shannon’s diversities (*α*-diversity) for patients that did or did not show CDI recurrence across multiple time points (pre- and post-treatment and pre- and post-discharge). The only time point when there was a significant difference in Shannon’s diversity between recurrent and non-recurrent patients was pre-treatment

**Fig. 3:**
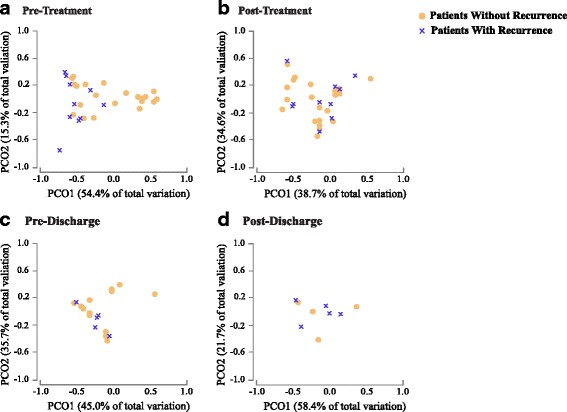
Principal Coordinate Analysis (PCoA) plots showing *β*-diversity differences between recurrent and non-recurrent patient samples at the pre-treatment (**a**), post-treatment (**b**), pre-discharge (**c**), and post-discharge (**d**) time points. The only time point when there was a significant difference in community structure (*β*-diversity) between recurrent and non-recurrent patients was pre-treatment

**Fig. 4:**
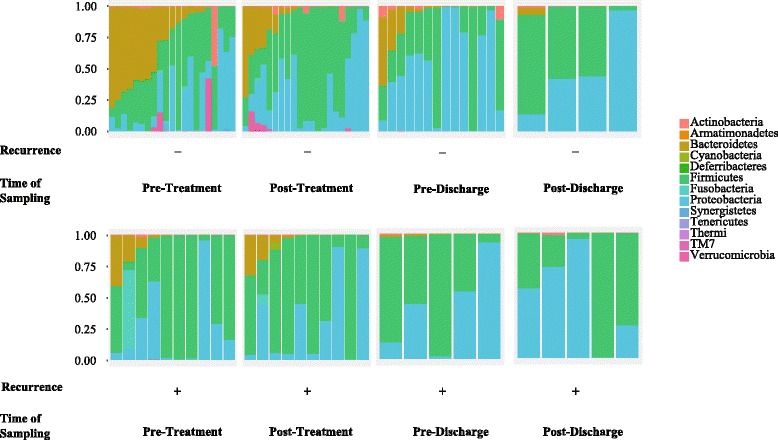
Relative abundances of bacterial phyla in recurrent vs. non-recurrent patients across the different sampling time points

**Fig. 5:**
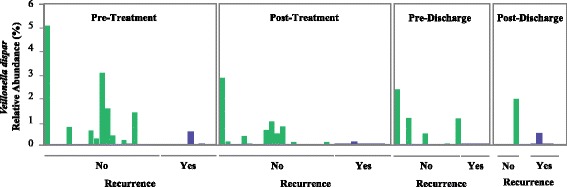
Bar plots show the relative abundance of *Veillonella dispar* OTU (predictive of CDI recurrence in our random forest model) in recurrent vs. non-recurrent patients across the different sampling time points. A significant difference in relative abundance of *Veilonella dispar* was observed between recurrent (*n* = 10) and non-recurrent (*n* = 21) patients (Mann-Whitney *U* test, adjusted *p* value = 0.026) at the pre-treatment time. All the *p* values were adjusted using the FDR correction

Our results surprisingly showed larger difference between patients (with and without recurrence) “before” treatment compared to after treatment. This is interesting because it could be clinically useful to identify which patient is more susceptible to recurrence. More specifically, when we compared microbial diversity and community structure of patients with and without recurrence, 16S rRNA gene data demonstrated a significant difference at the pre-treatment stage in *α*-diversity (measured by Shannon’s entropy) between these groups of patients (Mann-Whitney *U* test, *p* value = 0.026 (Fig. 2). Over the course of treatment, the difference between the two groups became marginal (Fig. 2). We observed a similar difference in *β*-diversity (weighted UniFrac; PERMANOVA, *p* value = 0.043) between the two groups at the pre-treatment stage (Fig. 3a), but not after treatment (Fig. 3b–d). At the phylum level, before treatment, patients with recurrence had lower abundance of Bacteroidetes than subjects without recurrence (Fig. 4). After treatment, the gut microbiota of both groups were dominated by Firmicutes and Proteobacteria (Fig. 4). At the individual OTU level, for the pre-treatment stage, results revealed a significant difference in relative abundance of *Veillonella dispar* (Mann-Whitney *U* test, adjusted *p* value = 0.026; Fig. 5). At the post-treatment and pre-discharge stages, the relative abundance of this species also was generally higher in patients without recurrence (Fig. 5), although these differences were not statistically significant. Even though at the genus and family levels *Veillonella* and Veillonellaceae were notably different between groups, they were found to be insignificant after multiple hypothesis correction.

To determine whether OTUs, genera, or families could serve as biomarkers to classify patients with or without recurrence at the pre-treatment stage, we constructed three separate random forest (RF) classifiers. The OTU-level RF had an error rate of 0.35 with the area under the ROC curve (AUC) of 0.68 (Additional file 2). The predictability of the OTU RF model was found to be significantly greater than randomly shuffled data (Wilcoxon signed-rank test, *p* value = 0.026). This model ranked OTUs belonging to *Veilonella dispar* as the most important variables for predicting recurrence (Additional file 2).

At the genus and family levels, error rates were 0.37 and 0.38, with the AUC of 0.57 and 0.53, respectively (Additional file 2). *Veillonella* ranked first at the genus level (Additional file 2), and Veillonellaceae ranked first at the family level (Additional file 2); however, the models’ predictabilities were found to be not significantly different from randomized data (Wilcoxon signed-rank test, *p* value > 0.05).

### Meta-analysis

We combined our high-throughput 16S rRNA gene sequence data with a recent study by Khanna et al. [22]. Although both studies shared a common experimental approach, results revealed a strong study effect as the most clearly discernible signal in the data (PERMANOVA, *p* value = 0.002). The random forest trained to classify which samples come from which study had an error rate of about 2% with AUC of 0.98 (Additional file 3). This resilient study-level effect was consistent, even when we included only shared OTUs between two studies. We then generated predictive models using our dataset with truncated sequences (200 bp, leave-one-out cross-validation), and the results showed a performance reduction at all taxonomic levels compared to our original dataset with sequence read lengths of 250 bp (Table 2). We also constructed three separate random forest (RF) classifiers of CDI recurrence using the Khanna et al. [22] dataset. Members of Veillonellaceae family were ranked first for all constructed models, albeit with no statistically significant discriminatory powers (Table 2). Finally, when we trained on our data and used the Khanna et al. [22] for cross-validation, the error rate was 0.29, and vice versa, the error rate was 0.32; none of these RF models were significant.

**Table 2:** Comparison of different random forest model predictions at three bacterial taxonomic levels

Study	Sequence length	Error rate	AUC	*p* value	Most important variable
C-OTU level	250	0.35	0.61	0.026	*Veillonella dispar*
C-Genus level	250	0.37	0.57	> 0.05	*Veillonella*
C-Family level	250	0.38	0.53	> 0.05	Veillonellaceae
C-OTU level	200	0.40	0.55	> 0.05	*Bacteroides uniformis*
C-Genus level	200	0.40	0.40	> 0.05	*Veillonella*
C-Family level	200	0.42	0.45	> 0.05	Veillonellaceae
K-OTU level	200	0.30	0.51	> 0.05	*Phascolarctobacterium* sp.
K-Genus level	200	0.34	0.46	> 0.05	*Phascolarctobacterium*
K-Family level	200	0.35	0.51	> 0.05	Veillonellaceae

*C* current study dataset, *K* Khanna et al.’s [22] dataset, *AUC* area under the ROC curve

## Discussion

Our results are in general agreement with the prior consensus that healthy and robust gut microbiota are protective against *C. difficile* invasion [37–39]—often termed “colonization resistance” [40, 41]. Disruption of the indigenous microbiota by perturbations, such as through the administration of antibiotics, can alter the overall physicochemical environment of the gut and the concentration of microbial and host metabolites [23, 26, 42, 43], as well as host immunity [44–46]. Such alterations can, in turn, yield lower colonization resistance and make the gut vulnerable to germination and toxin production by indigenous *C. difficile* or invasion by exogenous *C. difficile* spores. We found that there were significant differences in the initial (pre-treatment) microbial community structure between patients who exhibited CDI recurrence and those who did not. Administration of antibiotics to treat the initial *C. difficile* infection resulted in large-scale changes in the gut microbial community, which made recurrent and non-recurrent post-treatment microbial communities much more similar to one another than to the healthy, untreated controls. We did not find any notable associations between gut flora and the risk of recurrent CDI after antibiotic administration. We hypothesize that patients who did not show recurrence were able to recover towards an invasion-resistant community configuration [47] compared to patients who showed infection recurrence, but the exact configuration of this invasion-resistant state remains unclear. The development of this invasion-resistant state could be related to the positive feedback between intestinal bacteria and the intestinal mucosa. For example, Johansson et al. [46] demonstrated the profound effect of indigenous gut microbiota on the dynamics of mucus layer development. We speculate that low diversity microbiota in recurrent CDI subjects may lead to alteration in their intestinal mucosa, which in turn can negatively influence the host modulating effect of gut microbiota and lead to infection relapse after full recovery. The ability to recover to an invasion-resistant community can also depend upon 7a-dehydroxylase activity and subsequently higher conversion rates of primary bile salts to secondary bile salts, which are inhibitory to the germination of *C. difficile* spores and protect against CDI [21, 26]. Finally, invasion resistance may also be achieved by the recovery of indigenous clostridia, which may exclude *C. difficile* by saturating its available niche space in the gut [48].

We developed random forest classification models using the microbiota data at different levels of taxonomic resolution. The only significant model was at the OTU level, which was able to differentiate, albeit not very reliably, between individuals with and without recurrent CDI; classification did not improve when the microbiota results were combined with patients’ clinical metadata. Our RF analysis identified several OTUs related to *Veillonella dispar* as the most important features for predicting CDI recurrence. These OTUs were significantly enriched in non-recurrent patients. At genus and family levels, members of Veillonellaceae and Lachnospiraceae were the top-ranked RF features. Our results support prior work suggesting a positive association between members of the Lachnospiraceae family and colonization resistance against CDI [23, 49], several members of which are butyrate-producing, anaerobic bacteria. Butyric acid is known to strengthen colonic defensive barriers by elevating antimicrobial peptide levels (AMPs) and mucin production [50, 51]. Our meta-analysis showed that technical variation between studies overshadowed the biological variation. The lack of full consistency between the two studies may also be rooted in the difference in the average age or ethnicity of the two cohorts (Table 1). In addition, no significant feature or model prediction was observed using the truncated sequences from our original analysis (i.e., truncated in order to match sequence lengths from the Khanna study). This clearly implies the necessity for longer sequence (≥ 250 bps) reads for differentiating between closely related but distinct bacterial taxa and subsequently for CDI classification models.

## Conclusion

The present work showed that patients’ microbiota before antibiotic treatment can be predictive of disease relapse, but surprisingly, post-antibiotic microbial community is indistinguishable between patients that recur or not. While fecal microbiota transplantation (FMT) has been effective for CDI therapeutics, there is a widespread interest in designing microbial therapies that rely on pure cultures of bacteria and that target CDI recurrence with greater safety and efficacy. Such efforts require identification of the gut microbial species conferring invasion resistance against *C. difficile*. In our patient population of Afro-Caribbean descent, *Veillonella dispar* could be a candidate organism for negatively predicting CDI recurrence. However, this cannot yet be generalized to other patient populations with different demographic characteristics, signifying the need for larger cohort studies that include patients with diverse demographic characteristics.

## Additional files

Additional file 1: Clinical metadata for patient cohort. (DOCX 21 kb)

Additional file 2: Random Forest (RF) models were fit to pre-treatment microbiome data at the OTU, genus, and family levels. The strongest RF model was at the OTU level, with an ROC AUC of 0.61 (A). The strongest predictors for the OTU RF model were two Viellonella dispar OTUs (B). At the genus level, the ROC AUC was 0.57 (C) and the strongest predictor was the Viellonella genus (D). At the family level, the ROC AUC was 0.53 (E) and the strongest predictor was Veillonellaceae (F). (PDF 847 kb)

Additional file 3: A Random Forest model was able to classify samples according to study with very high accuracy (ROC AUC = 0.98). (PDF 745 kb)

## References

[1] Kuijper EJ, Coignard B, Tull P, ESCMID Study Group for Clostridium difficile, European Centre for Disease Prevention and Control (2006). Emergence of *Clostridium difficile*-associated disease in North America and Europe. Clin Microbiol Infect.

[2] Banaei N, Anikst V, Schroeder LF (2015). Burden of *Clostridium difficile* infection in the United States. N Engl J Med.

[3] Gravel D, Miller M, Simor A, Taylor G, Gardam M, McGeer A, Hutchinson J, Moore D, Kelly S, Boyd D (2009). Health care-associated *Clostridium difficile* infection in adults admitted to acute care hospitals in Canada: a Canadian nosocomial infection surveillance program study. Clin Infect Dis.

[4] Cheng JW, Xiao M, Kudinha T, Xu ZP, Hou X, Sun LY, Zhang L, Fan X, Kong FR, Xu YC. The first two *Clostridium difficile* ribotype 027/st1 isolates identified in Beijing, China—an emerging problem or a neglected threat? Sci Rep. 2016;6:1–8. 10.1038/srep18834 PMC4703979 26740150

[5] Jones AM, Kuijper EJ, Wilcox MH (2013). *Clostridium difficile*: a European perspective. J Infect.

[6] Kola A, Wiuff C, Akerlund T, van Benthem BH, Coignard B, Lyytikainen O, Weitzel-Kage D, Suetens C, Wilcox MH, Kuijper EJ (2016). Survey of *Clostridium difficile* infection surveillance systems in Europe, 2011. Eur Secur.

[7] Kelly CP, LaMont JT. *Clostridium difficile*—more difficult than ever. N Engl J Med. 2008;359 10.1056/NEJMra0707500 18971494

[8] Dallal RM, Harbrecht BG, Boujoukas AJ, Sirio CA, Farkas LM, Lee KK, Simmons RL (2002). Fulminant *Clostridium difficile*: an underappreciated and increasing cause of death and complications. Ann Surg.

[9] Forster AJ, Taljaard M, Oake N, Wilson K, Roth V, van Walraven C (2012). The effect of hospital-acquired infection with *Clostridium difficile* on length of stay in hospital. Can Med Assoc J.

[10] Khanna S, Gupta A, Baddour LM, Pardi DS (2016). Epidemiology, outcomes, and predictors of mortality in hospitalized adults with *Clostridium difficile* infection. Intern Emerg Med.

[11] Khanna S, Pardi DS (2010). The growing incidence and severity of *Clostridium difficile* infection in inpatient and outpatient settings. Expert Review of Gastroenterology & Hepatology.

[12] Dos Santos-Schaller O, Boisset S, Seigneurin A, Epaulard O. Recurrence and death after *Clostridium difficile* infection: gender-dependant influence of proton pump inhibitor therapy. Spring. 2016;5:1–5. 10.1186/s40064-016-2058-z PMC4828342 27104118

[13] Kwok CS, Arthur AK, Anibueze CI, Singh S, Cavallazzi R, Loke YK (2012). Risk of *Clostridium difficile* infection with acid suppressing drugs and antibiotics: meta-analysis. Am J Gastroenterol.

[14] Khanna S, Pardi DS (2012). *Clostridium difficile* infection: new insights into management. Mayo Clin Proc.

[15] Khanna S, Pardi DS (2014). *Clostridium difficile* infection: management strategies for a difficult disease. Ther Adv Gastroenterol.

[16] Vardakas KZ, Polyzos KA, Patouni K, Rafailidis PI, Samonis G, Falagas ME (2012). Treatment failure and recurrence of Clostridium difficile infection following treatment with vancomycin or metronidazole: a systematic review of the evidence. Int J Antimicrob Agents.

[17] McDonald LC, Coignard B, Dubberke E, Song XY, Horan T, Kutty PK, Ad Hoc Clostridium Difficile S (2007). Recommendations for surveillance of *Clostridium difficile* associated disease. Infect Control Hosp Epidemiol.

[18] Shivashankar R, Khanna S, Kammer PP, Harmsen WS, Zinsmeister AR, Baddour LM, Pardi DS (2013). Clinical factors associated with development of severe-complicated *Clostridium difficile* infection. Clin Gastroenterol Hepatol.

[19] Zhang FM, Luo WS, Shi Y, Fan ZN, Ji GZ. Should we standardize the 1,700-year-old fecal microbiota transplantation? Am J Gastroenterol. 2012;107:1755–5. 10.1038/ajg.2012.251 23160295

[20] Guo B, Harstall C, Louie T, van Zanten SV, Dieleman LA (2012). Systematic review: faecal transplantation for the treatment of *Clostridium difficile*-associated disease. Aliment Pharmacol Ther.

[21] Theriot CM, Koenigsknecht MJ, Carlson PE, Hatton GE, Nelson AM, Li B, Huffnagle GB, Li JZ, Young VB. Antibiotic-induced shifts in the mouse gut microbiome and metabolome increase susceptibility to *Clostridium difficile* infection. Nat Commun. 2014;5:1–10. 10.1038/ncomms4114 PMC3950275 24445449

[22] Khanna S, Montassier E, Schmidt B, Patel R, Knights D, Pardi DS, Kashyap PC (2016). Gut microbiome predictors of treatment response and recurrence in primary *Clostridium difficile* infection. Aliment Pharmacol Ther.

[23] Antharam VC, Li EC, Ishmael A, Sharma A, Mai V, Rand KH, Wang GP (2013). Intestinal dysbiosis and depletion of butyrogenic bacteria in *Clostridium difficile* infection and nosocomial diarrhea. J Clin Microbiol.

[24] Chang JY, Antonopoulos DA, Kalra A, Tonelli A, Khalife WT, Schmidt TM, Young VB (2008). Decreased diversity of the fecal microbiome in recurrent *Clostridium difficile*-associated diarrhea. J Infect Dis.

[25] Schubert AM, Rogers MAM, Ring C, Mogle J, Petrosino JP, Young VB, Aronoff DM, Schloss PD. Microbiome data distinguish patients with *Clostridium difficile* infection and non-C. difficile-associated diarrhea from healthy controls. MBio. 2014;5:1–9. 10.1128/mBio.01021-14 PMC4010826 24803517

[26] Allegretti JR, Kearney S, Li N, Bogart E, Bullock K, Gerber GK, Bry L, Clish CB, Alm E, Korzenik JR (2016). Recurrent Clostridium difficile infection associates with distinct bile acid and microbiome profiles. Aliment Pharmacol Ther.

[27] Seekatz AM, Young VB (2014). *Clostridium difficile* and the microbiota. J Clin Investig.

[28] Antharam VC, McEwen DC, Garrett TJ, Dossey AT, Li EC, Kozlov AN, Mesbah Z, Wang GP. An integrated metabolomic and microbiome analysis identified specific gut microbiota associated with fecal cholesterol and coprostanol in *clostridium difficile* infection. PLoS One. 2016;11:1–23. 10.1371/journal.pone.0148824 PMC4752508 26871580

[29] Gujja D, Friedenberg FK (2009). Predictors of serious complications due to Clostridium difficile infection. Aliment Pharmacol Ther.

[30] McEllistrem MC, Carman RJ, Gerding DN, Genheimer CW, Zheng L (2005). A hospital outbreak of Clostridium difficile disease associated with isolates carrying binary toxin genes. Clin Infect Dis.

[31] Caporaso JG, Kuczynski J, Stombaugh J, Bittinger K, Bushman FD, Costello EK, Fierer N, Pena AG, Goodrich JK, Gordon JI (2010). QIIME allows analysis of high-throughput community sequencing data. Nat Methods.

[32] Edgar RC. UPARSE: highly accurate OTU sequences from microbial amplicon reads. Nat Methods. 2013;10:996–98. 10.1038/nmeth.2604 23955772

[33] Edgar RC. Search and clustering orders of magnitude faster than BLAST. Bioinformatics. 2010;26:2460–2461. 10.1093/bioinformatics/btq461 20709691

[34] McDonald D, Price MN, Goodrich J, Nawrocki EP, DeSantis TZ, Probst A, Andersen GL, Knight R, Hugenholtz P (2012). An improved Greengenes taxonomy with explicit ranks for ecological and evolutionary analyses of bacteria and archaea. ISME J.

[35] Caporaso JG, Bittinger K, Bushman FD, DeSantis TZ, Andersen GL, Knight R (2010). PyNAST: a flexible tool for aligning sequences to a template alignment. Bioinformatics.

[36] Price MN, Dehal PS, Arkin AP. FastTree 2-approximately maximum-likelihood trees for large alignments. PLoS One. 2010;5 10.1371/journal.pone.0009490 PMC2835736 20224823

[37] Wilson KH (1993). The microecology of Clostridium difficile. Clin Infect Dis.

[38] Sorg JA, Sonenshein AL (2008). Bile salts and glycine as cogerminants for *Clostridium difficile* spores. J Bacteriol.

[39] Buffie CG, Bucci V, Stein RR, McKenney PT, Ling LL, Gobourne A, No D, Liu H, Kinnebrew M, Viale A (2015). Precision microbiome reconstitution restores bile acid mediated resistance to *Clostridium difficile*. Nature.

[40] Vanderwaaij D (1987). Colonization resistance of the digestive tract—mechanism and clinical consequences. Nahrung/Food.

[41] Van der Waaij D, Berghuis-de Vries JM, Lekkerkerk-van der Wees JEC (1971). Colonization resistance of the digestive tract in conventional and antibiotic-treated mice. J Hyg.

[42] Wilson M (2005). Microbial inhabitants of humans: their ecology and role in health and disease.

[43] Yap IKS, Li JV, Saric J, Martin FP, Davies H, Wang YL, Wilson ID, Nicholson JK, Utzinger J, Marchesi JR, Holmes E (2008). Metabonomic and microbiological analysis of the dynamic effect of vancomycin-induced gut microbiota modification in the mouse. J Proteome Res.

[44] Wlodarska M, Willing B, Keeney KM, Menendez A, Bergstrom KS, Gill N, Russell SL, Vallance BA, Finlay BB (2011). Antibiotic treatment alters the colonic mucus layer and predisposes the host to exacerbated Citrobacter rodentium-induced colitis. Infect Immun.

[45] Ghosh S, Dai C, Brown K, Rajendiran E, Makarenko S, Baker J, Ma C, Halder S, Montero M, Ionescu VA (2011). Colonic microbiota alters host susceptibility to infectious colitis by modulating inflammation, redox status, and ion transporter gene expression. American Journal of Physiology-Gastrointestinal and Liver Physiology.

[46] Johansson MEV, Jakobsson HE, Holmen-Larsson J, Schutte A, Ermund A, Rodriguez-Pineiro AM, Arike L, Wising C, Svensson F, Backhed F, Hansson GC (2015). Normalization of host intestinal mucus layers requires long-term microbial colonization. Cell Host Microbe.

[47] Fischbach MA, Segre JA (2016). Signaling in host-associated microbial communities. Cell.

[48] Bien J, Palagani V, Bozko P (2013). The intestinal microbiota dysbiosis and Clostridium difficile infection: is there a relationship with inflammatory bowel disease?. Ther Adv Gastroenterol.

[49] Reeves AE, Koenigsknecht MJ, Bergin IL, Young VB (2012). Suppression of *clostridium difficile* in the gastrointestinal tracts of germfree mice inoculated with a murine isolate from the family lachnospiraceae. Infect Immun.

[50] Cook SI, Sellin JH (1998). Review article: short chain fatty acids in health and disease. Aliment Pharmacol Ther.

[51] Wong JMW, de Souza R, Kendall CWC, Emam A, Jenkins DJA (2006). Colonic health: fermentation and short chain fatty acids. J Clin Gastroenterol.

