# Supplementary material for: Identifying predictive features of Clostridium difficile infection recurrence before, during, and after primary antibiotic treatment
Source: Microbiome. 2017 Nov 13;5:148. doi: 10.1186/s40168-017-0368-1 (PMC5684761; doi:10.1186/s40168-017-0368-1)
Supplement: Supplementary file 1 — Clinical metadata for patient cohort. (DOCX 21 kb) [file 40168_2017_368_MOESM1_ESM.docx]

**Supplementary Document 1:**

|  | | | |  |  |  |  |  |  |  |  |  |  |
| --- | --- | --- | --- | --- | --- | --- | --- | --- | --- | --- | --- | --- | --- |
| **Table S1.** Clinical metadata for patient cohort. | | | | | | | | | | | | | |
|  | | | |  |  |  |  |  |  |  |  |  |  |
| Pt ID | AGE | SEX | Race | BMI | Recent ABX Used ᶠ | PPI/H2 Use ᶣ | Tmax (F) | Abdominal Pain and/or distension | WBC* | Positive CT Findings^ | UMPC Score ᶨ | Recurrence |  |
| 1 | 60 | M | Black | 15.17 | Vanc, Zosyn, Flagyl, Cipro | Esomeprazole | 98.4 | Yes | No | Yes | 2 | No |  |
| 2 | 71 | M | Black | 26.4 | Vanc, Zosyn | Esomeprazole | 97.2 | No | No | No | 0 | Yes |  |
| 3 | 62 | M | Black | 20.82 | None | No | 98.8 | Yes | No | No | 1 | No |  |
| 4 | 62 | M | Black | 40.76 | Vanc, Zosyn, Flagyl | Esomeprazole | 102.4 | Yes | No | No | 1 | Yes |  |
| 5 | 65 | M | Black | 23.05 | None | No | 98.8 | No | No | No | 0 | No |  |
| 6 | 48 | M | Black | 38.42 | Cipro, Ceph | Esomeprazole | 99.8 | Yes | No | No | 1 | Yes |  |
| 7 | 79 | F | White | 20.38 | Flagyl, Cipro | Famotidine | 99.9 | Yes | No | No | 2 | Yes |  |
| 8 | 73 | M | White | 23.69 | Vanc, Flagyl | Pantoprazole | 99.5 | Yes | No | Yes | 2 | No |  |
| 9 | 78 | F | Black | 27.26 | Zosyn | No | 97.2 | Yes | No | No | 1 | Yes |  |
| 10 | 71 | F | Black | 22.49 | Vanc, Zosyn | No | 99.9 | Yes | No | Yes | 1 | No |  |
| 11 | 32 | M | Black | 19.51 | None | Esomeprazole | 99.7 | No | No | No | 0 | Yes |  |
| 12 | 62 | F | Black | 23.49 | Vanc, Flagyl | Pantoprazole | 99.8 | No | No | No | 0 | No |  |
| 13 | 80 | F | Black | 19.75 | None | No | 99.8 | No | No | No | 1 | No |  |
| 14 | 83 | M | Black | 35.68 | Vanc | No | 98.8 | No | No | No | 0 | No |  |
| 15 | 91 | F | Black | 31.67 | None | Esomeprazole | 101.6 | Yes | No | Yes | 1 | No |  |
| 16 | 36 | F | Black | 18.08 | None | No | 99.8 | Yes | No | No | 1 | No |  |
| 17 | 52 | F | Black | 36.39 | None | Esomeprazole | 100.2 | Yes | No | No | 2 | Yes |  |
| 18 | 74 | F | Black | 19.95 | Vanc, Zosyn | Famotidine | 98.8 | No | No | No | 0 | No |  |
| 19 | 68 | F | White | 16.37 | Vanc, Zosyn | Pantoprazole | 99.8 | Yes | Yes | Yes | 3 | No |  |
| 20 | 60 | M | Black | UNK | None | Esomeprazole | 97.2 | No | Yes | No | 1 | No |  |
| 21 | 43 | M | Black | 21.22 | None | No | 97.8 | No | No | Untested | 0 | No |  |
| 22 | 53 | F | Black | 36.15 | Vanc, Zosyn, Flagyl, Cipro | Esomeprazole | 101.8 | No | No | Yes | 0 | No |  |
| 23 | 64 | F | Black | 30.41 | Cipro, Ceph | No | 98.2 | No | No | No | 0 | No |  |
| 24 | 41 | M | Black | 31.74 | Vanc, Zosyn | Esomeprazole | 98.8 | No | No | No | 0 | Yes |  |
| 25 | 63 | M | Black | 25.03 | None | Esomeprazole | 98.8 | No | No | No | 0 | Yes |  |
| 26 | 60 | F | Black | 26.36 | Ceph | Esomeprazole | 98 | No | No | No | 0 | No |  |
| 27 | 65 | F | Black | 33.18 | Vanc, Zosyn, Cipro, Ceph | Esomeprazole | 99.8 | Yes | No | No | 2 | No |  |
| 28 | 76 | M | Black | 24.99 | Ceph | Famotidine | 99.8 | Yes | Yes | No | 3 | No |  |
| 29 | 71 | F | Black | 30.07 | Ceph | Esomeprazole | 98.6 | No | No | No | 0 | Yes |  |
| 30 | 77 | M | Black | 28.43 | Vanc | No | 98.6 | No | Yes | No | 1 | No |  |
| 31 | 72 | F | Black | 14.4 | Zosyn | No | 98.1 | No | No | No | 0 | No |  |

ᶠ Recent used antibiotics within the past 3 month; (Vanc) Vancomycin, (Cipro) Ciprofloxacin, (Zosyn) Piperacillin and Taxobactam, (Flagyl) Metronidazole, and (Ceph) Cephalexin.

ᶣ Proton Pump Inhibitor

** Does patient have WBC count over 20x10^9/L or below 1.5 x 10^9/L or WBC bands greater than 10%?

^^ Does the patient have CT scan findings of colitis, pneumatosis coli, or bowel wall thickening?

ᶨ University of Pittsburgh Medical Center Severity Classification (scored 0-3).

**Table S2.** Patient stool samples collected for microbiome sequencing.

| Pt ID | Pre-Treatment | Post-Treatment | Pre-Discharge | Post-Discharge |
| --- | --- | --- | --- | --- |
| 1 | X | X | X | - |
| 2 | X | X | X | X |
| 3 | X | X | X | - |
| 4 | X | X | X | - |
| 5 | X | X | - | - |
| 6 | X | X | - | - |
| 7 | X | X | X | X |
| 8 | X | X | - | - |
| 9 | X | X | X | X |
| 10 | X | X | X |  |
| 11 | X | X | X | X |
| 12 | X | X | X | X |
| 13 | X | X | X | - |
| 14 | X | X | - | - |
| 15 | X | X | X | - |
| 16 | X | X | - | - |
| 17 | X | X | - | - |
| 18 | X | X | X |  |
| 19 | X | X | - | - |
| 20 | X | X | X | - |
| 21 | X | X | - | - |
| 22 | X | X | - | - |
| 23 | X | X | - | - |
| 24 | X | X | - | - |
| 25 | X | X | - | X |
| 26 | X | X | X | - |
| 27 | X | X | X | X |
| 28 | X | X | X | X |
| 29 | X | X | - | - |
| 30 | X | X | X | X |
| 31 | X | X | X | - |

(Pre-treatment) samples at patient admission; post-treatment, (Post-Treatment) sample collected 2 says after beginning treatment for CDiff colitis, (Pre-Discharge) samples collected 7 days after the start of antibiotics treatment or at the time of discharge, (Post-Discharge) two weeks after the start of antibiotics.
