# Supplementary material for: Identifying predictive features of Clostridium difficile infection recurrence before, during, and after primary antibiotic treatment
Source: Microbiome. 2017 Nov 13;5:148. doi: 10.1186/s40168-017-0368-1 (PMC5684761; doi:10.1186/s40168-017-0368-1)
Supplement: Supplementary file 2 — Random Forest (RF) models were fit to pre-treatment microbiome data at the OTU, genus, and family levels. The strongest RF model was at the OTU level, with an ROC AUC of 0.61 (A). The strongest predictors for the OTU RF model were two Viellonella dispar OTUs (B). At the genus level, the ROC AUC was 0.57 (C) and the strongest predictor was the Viellonella genus (D). At the family level, the ROC AUC was 0.53 (E) and the strongest predictor was Veillonellaceae (F). (PDF 847 kb) [file 40168_2017_368_MOESM2_ESM.pdf]

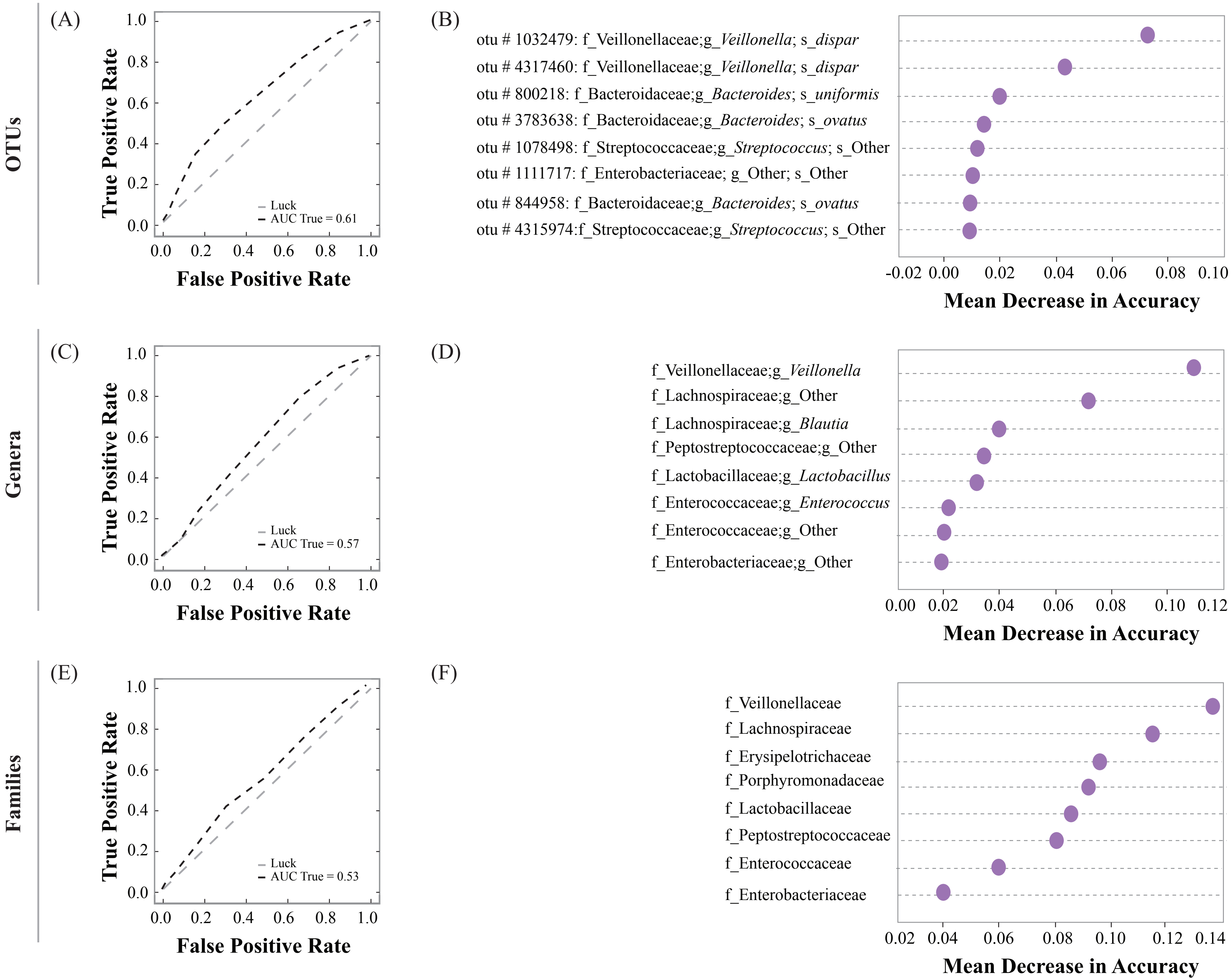

OTUs
Genera
Families
(A)
(B)
(C)
(D)
(E)
(F)
True Positive Rate
False Positive Rate
Luck
AUC True = 0.61
AUC True = 0.57
AUC True = 0.53
Mean Decrease in Accuracy
otu # 1032479: f_Veillonellaceae;g_Veillonella; s_dispar
otu # 4317460: f_Veillonellaceae;g_Veillonella; s_dispar
otu # 800218: f_Bacteroidaceae;g_Bacteroides; s_uniformis
otu # 3783638: f_Bacteroidaceae;g_Bacteroides; s_ovatus
otu # 1078498: f_Streptococcaceae;g_Streptococcus; s_Other
otu # 1111717: f_Enterobacteriaceae; g_Other; s_Other
otu # 844958: f_Bacteroidaceae;g_Bacteroides; s_ovatus
otu # 4315974:f_Streptococcaceae;g_Streptococcus; s_Other
f_Veillonellaceae;g_Veillonella
f_Lachnospiraceae;g_Other
f_Lachnospiraceae;g_Blautia
f_Peptostreptococcaceae;g_Other
f_Lactobacillaceae;g_Lactobacillus
f_Enterococcaceae;g_Enterococcus
f_Enterococcaceae;g_Other
f_Enterobacteriaceae;g_Other
f_Veillonellaceae
f_Lachnospiraceae
f_Erysipelotrichaceae
f_Porphyromonadaceae
f_Lactobacillaceae
f_Peptostreptococcaceae
f_Enterococcaceae
f_Enterobacteriaceae
