# Supplementary figures and images for: Identifying predictive features of Clostridium difficile infection recurrence before, during, and after primary antibiotic treatment
Source: Microbiome. 2017 Nov 13;5:148. doi: 10.1186/s40168-017-0368-1 (PMC5684761; doi:10.1186/s40168-017-0368-1)

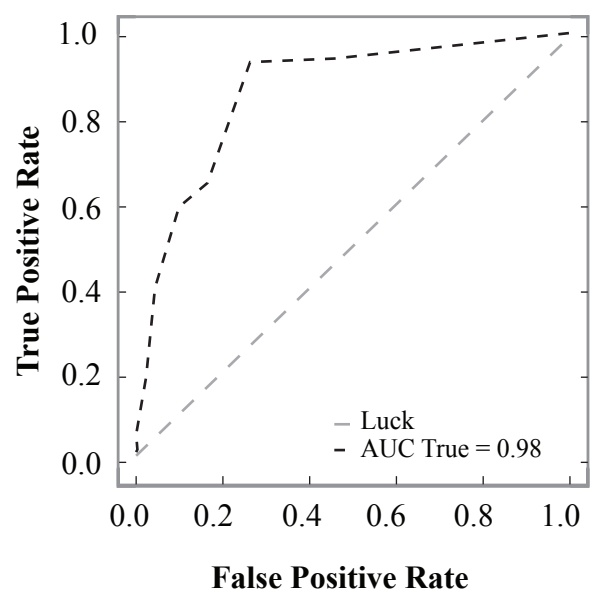

Supplement: Supplementary file 3 — A Random Forest model was able to classify samples according to study with very high accuracy (ROC AUC = 0.98). (PDF 745 kb) [file 40168_2017_368_MOESM3_ESM.pdf]
